# Development and Characterization of a Patient-Derived *KIF5B-RET* Fusion Lung Adenocarcinoma Cell Line for Subcutaneous and Central Nervous System Tumor Models in Immunodeficient Mice

**DOI:** 10.1016/j.jtocrr.2026.101026

**Published:** 2026-06-01

**Authors:** Akihiro Nishiyama, Shigeki Sato, Hiroyuki Sakaguchi, Hiroshi Kotani, Kaname Yamashita, Koushiro Ohtsubo, Shigeki Nanjo, Seiji Yano, Keishi Mizuguchi, Hiroko Ikeda, Makiko Yamaguchi, Tamotsu Ishizuka, Satoko Baba, Kengo Takeuchi, Shinji Takeuchi

**Affiliations:** a Department of Medical Oncology, Kanazawa University Hospital, Kanazawa, Japan; b Department of Respiratory Medicine, Kanazawa University Hospital, Kanazawa, Japan; c Department of Diagnostic Pathology, Kanazawa University Hospital, Kanazawa, Japan; d Department of Respiratory Medicine, Faculty of Medical Sciences, University of Fukui, Eiheiji, Japan; e Division of Pathology, Cancer Institute, Pathology Project for Molecular Targets, Cancer Institute, Japanese Foundation for Cancer Research, Tokyo, Japan; f Department of Pathology, Cancer Institute Hospital, Japanese Foundation for Cancer Research, Tokyo, Japan

**Keywords:** *KIF5B-RET* fusion, Patient-derived cell line, Subcutaneous tumor model, Central nervous system disease model, Selective *RET* tyrosine kinase inhibitor

## Abstract

**Introduction:**

This study presents the development and characterization of a novel lung adenocarcinoma cell line with kinesin family member 5B rearranged during transfection (*KIF5B-RET*) fusion, derived from a single patient's pleural effusion.

**Methods:**

A pleural effusion sample was collected from a patient with *RET* fusion-positive NSCLC. The cell line was developed from the pleural effusion, and its characteristics were investigated in vitro and in vivo. Information on lung cancer cell lines with *RET* fusions was collected through a literature review. The characteristics of the *RET* fusion-positive cell line developed were then compared with information on *RET* fusion-positive cell lines identified in the literature.

**Results:**

The newly established PB302 cell line has the following three key characteristics: high sensitivity to selective *RET* inhibitors, the ability to grow in the central nervous system, and the ability to grow subcutaneously in immunodeficient mice. These features enabled the development of central nervous system disease and subcutaneous tumor models. A comparative analysis revealed that PB302 uniquely possesses these characteristics among *RET*-positive lung adenocarcinoma cell lines.

**Conclusions:**

This cell line highlights several potentials as a tool for preclinical research and drug development.

## Introduction

The *RET* gene was first discovered by Dr. Takahashi in 1985.^1^ *RET* is activated when glial cell line–derived neurotrophic factor binds to the glial cell line–derived neurotrophic factor family receptor co-receptor, whereas calcium ions simultaneously bind to the cadherin-like extracellular domain of *RET*. This dual binding recruits *RET* and forms the *RET–GFR* complex,^2^ bringing two *RET* monomers together and inducing homodimerization and tyrosine kinase domain phosphorylation. Oncogenic *RET* activation can occur through chromosomal rearrangements involving *RET* and a gene partner with a dimerization domain, such as a coiled-coil domain. These fusions cause ligand-independent phosphorylation and constitutive *RET* activation.^3^

*RET* fusions occur in approximately 2% of patients with NSCLC, most often involving *KIF5B*.^4^^,^^5^ Approximately 20% of patients with *RET* fusion-positive lung cancer present with central nervous system (CNS) lesions.^6^ The selective *RET* tyrosine kinase inhibitor (TKI), selpercatinib, has demonstrated consistently positive clinical trial results, leading to approval by the U.S. Food and Drug Administration for treating *RET* fusion-positive solid tumors, including NSCLC.^7^, ^8^, ^9^ Selpercatinib binds to the adenosine pocket between K758 and V804 in the *RET* tyrosine kinase domain, competing with ATP to inhibit its activity.^10^ Selpercatinib and another selective *RET* TKI, pralsetinib, demonstrate high response rates for extracranial and cranial lesions. However, resistance to selective *RET* inhibitors occurs in approximately 4% of cases.^6^ Thus, acquired drug resistance remains a significant clinical challenge.^11^ Next-generation *RET* inhibitors are being developed to treat *RET* solvent-front mutations.

Addressing these clinical challenges requires preclinical experiments using cell lines. Several *RET* fusion-positive adenocarcinoma cell lines, including the commercially available LC-2/ad, have been reported, each with distinct characteristics.^12^, ^13^, ^14^ A more versatile cell line is needed to provide a robust model for subcutaneous tumors and CNS lesions.

## Materials and Methods

### Cell Lines and Reagents

The pleural mesothelioma cell line EHMES-10 harboring the *NCOA4-RET* fusion from the Tokushima University, the human lung adenocarcinoma cell line LC-2/ad harboring the *CCDC6-RET* fusion from the RIKEN BioResource Research Center, the wild-type human lung adenosquamous carcinoma cell line HCC366 from the DSMZ—German Collection of Microorganisms and Cell Cultures, and the wild-type human lung adenocarcinoma cell line H838 from the American Type Culture Collection were used. These cell lines were maintained in RPMI-1640 medium supplemented with 10% fetal bovine serum (FBS), penicillin (100 U/mL), and streptomycin (10 μg/mL) and cultured in a humidified CO_2_ incubator at 37°C. Cells were passaged for less than 3 months before renewal from frozen early passage stocks. Mycoplasma contamination was regularly screened using the MycoAlert Mycoplasma Detection Kit (Lonza, Rockland, ME). Selpercatinib, pralsetinib, and pemetrexed were purchased from Selleck Chemicals (Houston, TX). Carboplatin was purchased from Fujifilm Wako Pure Chemical Corporation (Osaka, Japan).

### Generation of the Patient-Derived Cell Line

**Figure 1:**
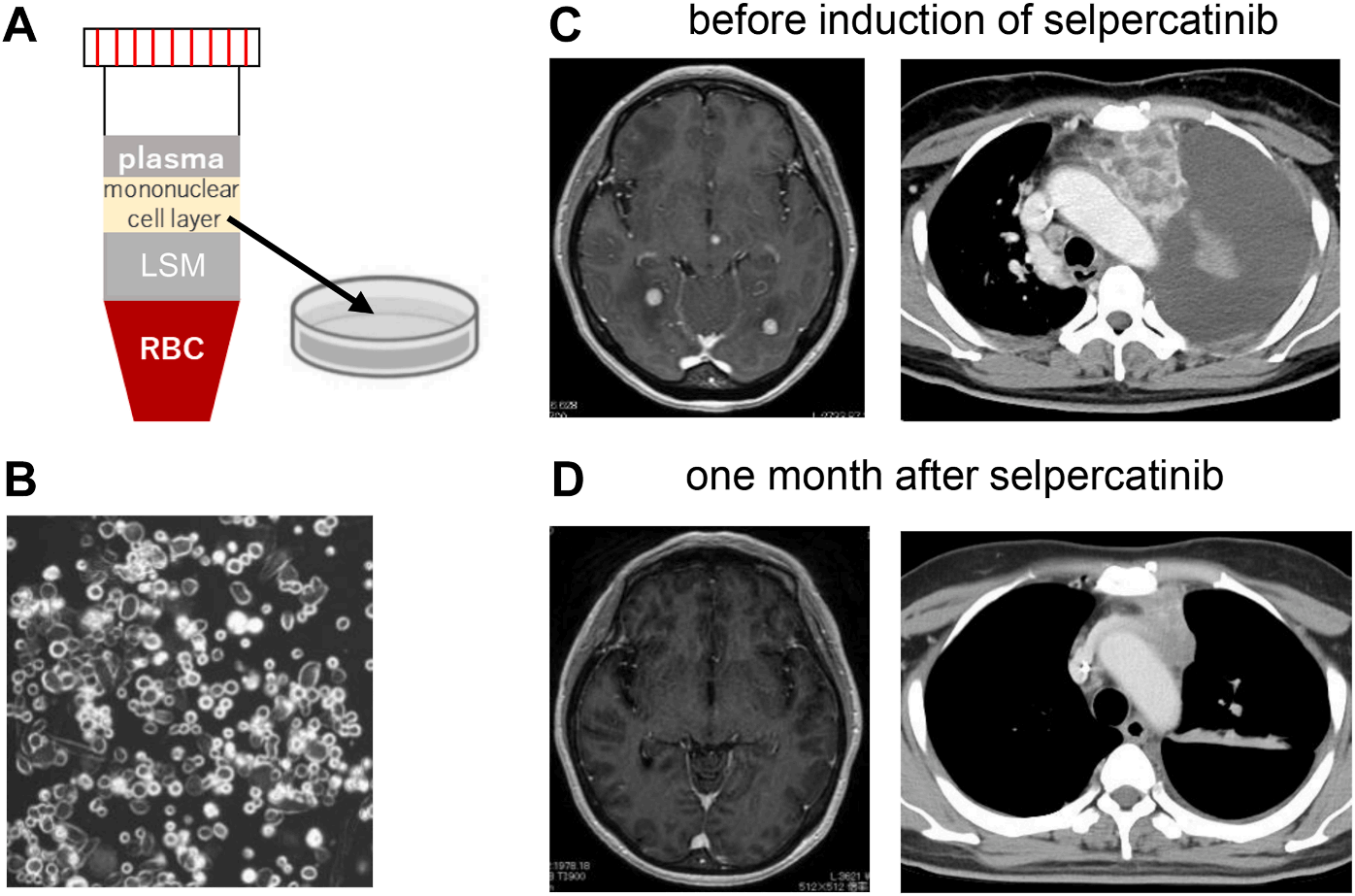
The cancer cell line PB302 was established from a single pleural effusion collected from a patient with *RET* fusion before the induction of selpercatinib. (*A*) Collection of cancer cells from the mononuclear cell layer using LSM. (*B*) Appearance of the established cell line PB302. (*C*) Leptomeningeal carcinomatosis and extensive pleural effusion before drug treatment. (*D*) Drastic improvement in imaging findings 1 month after starting selpercatinib. LSM, lymphocyte separation medium.

With patient consent, pleural effusion was collected under a biospecimen protocol approved by the Institutional Review Board (Kanazawa), Japan. Pleural effusion was obtained via thoracentesis before treatment was initiated with the investigated drug. The sample was centrifuged at 400 ×g for 15 minutes in lymphocyte separation medium. Cells from the mononuclear layer were collected using a 1000 μL micropipette and resuspended in RPMI-1640 medium supplemented with 10% FBS, penicillin (100 U/mL), and streptomycin (10 μg/mL) (Fig. 1*A*). The medium was replaced twice weekly, and cells were passaged three times, yielding a relatively uniform morphology. After three successive passages, the cell line was established and designated PB302 (Fig. 1*B*).

### EGFP-Eluc Gene Transfection

The expression vector EGFP-Eluc (containing Enhanced Green Fluorescent Protein [EGFP] from the pIRES-EGFP Vector and Emerald Luc [Eluc] from the Emerald Luc Vector)^15^ was transfected into PB302 cells using Lipofectamine LTX (Invitrogen) according to the manufacturer's protocol. Transfected cells were selected using puromycin.

### RNA Interference

Duplexed Silencer Select siRNAs targeting *RET* (s11935 and s11936) and Silencer Select Negative Control #1 siRNA (Ambion) were used for RNA interference assays. Briefly, 1 × 10^5^ cells were plated in 2 mL of antibiotic-free medium per well of a 6-well plate and incubated at 37°C for 24 hours. Cells were then transfected with 250 pmol siRNA or scrambled RNA using 5 μL of Lipofectamine RNAiMAX (Invitrogen) according to the manufacturer's protocol. After 24 hours, cells were washed twice with phosphate-buffered saline and incubated for an additional 48 hours in antibiotic-containing medium. These cells were used for cell proliferation assays, and *RET* knockdown was confirmed by Western blot.

### RNA Sequencing

Total RNA was extracted from PB302 cells using the QIAGEN RNeasy Mini Kit. mRNA was isolated using the poly(A) mRNA Magnetic Isolation Module (New England Biolabs, Ipswich, MA). Library preparation was performed with the NEBNext Ultra II Directional RNA Library Prep Kit for Illumina (New England Biolabs) according to the NEB protocol (https://international.neb.com/protocols/2014/12/01/protocol-for-use-with-nebnext-poly-a-mrna-magnetic-isolation-module-neb-e7490). Sequencing was conducted using the NovaSeq 6000 platform (Illumina Inc., San Diego, CA). Raw paired-end reads were quality checked using FastQC (version 0.11.7; https://www.bioinformatics.babraham.ac.uk/projects/fastqc/), and low-quality bases (<20) were filtered. Adapter sequences were trimmed using Trimmomatic (version 0.38) with the parameters: ILLUMINACLIP:path/to/adapter. fa:2:30:10 LEADING:20 TRAILING:20 SLIDINGWINDOW:4:15 MINLEN:36. Trimmed reads were aligned to the reference genome using STAR (version 2.7.4), and gene fusions were detected using Arriba (version 1.2.0). All analyses were performed by Rhelixa Co., Ltd.

### Whole Genome Sequencing Analysis

PB302 DNA was extracted using the Maxwell RSC Blood DNA Kit (Promega), and sample quality was assessed. Although the kit name includes “Blood,” it is not limited to blood samples and can purify genomic DNA from various biological materials, including cultured cells and tissues. The kit uses a purification principle that combines cell lysis with proteinase K and guanidine-based buffers with magnetic bead-based purification in the Maxwell automated nucleic acid purification system.

This approach is applicable to cultured cells and has been widely used across different sample types.^16^ Therefore, its use for genomic DNA extraction from cultured cells in this study was considered appropriate. Sequencing was performed using the NovaSeq X Plus (Illumina). For sample pretreatment, libraries were prepared with the TruSeq DNA polymerase chain reaction–free library prep kit (Illumina) and sequenced using Illumina flow cells. Bioinformatic analysis followed this workflow: (1) read quality was evaluated using FastQC and MultiQC; (2) adapter sequences were removed using Dynamic Read Analysis for GENomics (DRAGEN); (3) reads were mapped to the reference genome using DRAGEN; (4) mapping quality was verified; (5) reads suitable for SNV/Del/SV/CNV detection were selected based on paired-end information and polymerase chain reaction duplication; and (6) single-nucleotide variants and structural variants (SNV/Del/SV/CNV) were detected using DRAGEN and annotated. All analyses were conducted by Riken Genesis Co., Ltd.

### Cell Block Preparation Method (Formalin Layered Method)

Cells, polyethylene (PE) tubes (1.5 mL), 10% buffered formalin, pathology embedding cassettes, embedding sponges, cutters, and cutting knives were prepared. Cultured cells were washed with phosphate-buffered saline, and the cell count was adjusted to approximately 1 × 10^6^ cells to facilitate processing. First, phosphate-buffered saline–washed cells were transferred to a 1.5 mL tube. Second, the tube was centrifuged, and the supernatant was removed. Third, 10% buffered formalin was slowly added, and the sample was fixed overnight. Fourth, the supernatant was decanted, and the tube was cut with a sharp blade. Fifth, a sponge was placed in a pathology cassette, the tube was inserted, the lid was closed, and the cassette was placed in a paraffin impregnation machine. Finally, after paraffin embedding, the solidified cells were scraped from the tube with a small spoon and embedded.

### Fusion FISH Assay

Prepared cell blocks were cut to 4 μm in thickness for hematoxylin and eosin staining and fluorescein in situ hybridization (FISH). The FISH procedure was described previously.^17^ FISH screening for other kinases used bacterial artificial chromosome (BAC) clone-derived DNA probes. For *KIF5B* and *RET*, BAC clones *KIF5B* (RP11-460H18) and *RET* (RP11-351D16) were used.

Details are available in the Clone Registry of the National Center for Biotechnology Information (http://www.ncbi.nlm.nih.gov/genome/clone/). Hybridized slides were stained with 4,6-diamino-2-phenylindole and examined using a BX51 fluorescence microscope (Olympus, Tokyo, Japan).

### Cell Viability Assay

Cell viability was evaluated using the MTT [3-(4,5-dimethylthiazol-2-yl)-2,5-diphenyl tetrazolium] dye reduction method. Cells were seeded in 96-well plates at 2 × 10^3^ cells per 100 μL RPMI-1640 medium containing 10% FBS and 1% PS and incubated overnight. On the following day, the indicated compound was added at varying concentrations (0, 0.001, 0.003, 0.01, 0.03, 0.1, 0.3, 1, 3, 10 μmol/L), and the cells were incubated for 72 hours. Then, 50 μg of MTT solution (2 mg/mL, Sigma, St. Louis, MO) was added to each well, and the cells were incubated for 2 hours. The medium was removed, and the dark blue formazan crystals were dissolved in 100 μL DMSO. Absorbance was measured using a microplate reader at 550 nm with a reference wavelength of 630 nm. Cell culture survival was expressed as a percentage relative to the untreated controls. MTT assays were performed in at least three independent biological experiments, with each condition tested in triplicate technical wells within each experiment.

### Antibodies and Western Blotting

Cells were lysed using Cell Lysis Buffer (Cell Signaling Technology) supplemented with 1% (v/v) Phosphatase Inhibitor Cocktail 3 (Sigma-Aldrich) and distilled water. Cell extracts (20 μg per lane) were separated by SDS-PAGE using Mini-PROTEAN TGX Precast Gels and transferred onto Immun-Blot PVDF membranes (Bio-Rad). The primary antibodies used were RET (D3D8R), phospho-RET (Tyr905), AKT, phospho-AKT (Ser473), β-actin, cleaved PARP ((Asp214) (D64E10) XP), and PARP (all from Cell Signaling Technology), and ERK1/ERK and phospho-ERK1/ERK2 (Thr202/Thr204) from R&D Systems. After primary antibody incubation, the membranes were incubated for 1 hour at room temperature with species-specific horseradish peroxidase–conjugated secondary antibodies. Bands were visualized using SuperSignal West Dura Extended Duration Substrate, an enhanced chemiluminescence reagent from Pierce Biotechnology.

### Animal Experiments

All animal experiments followed the Guide for the Care and Use of Laboratory Animals issued by the Ministry of Education, Culture, Sports, Science, and Technology of Japan. The protocol was approved by the Committee on Ethics of Experimental Animals and the Advanced Science Research Center at Kanazawa University (approval number: AP-214264). Five-week-old hairless male SCID (C. B-17/Icr-scid/scidJc) SHO mice were obtained from Charles River Laboratories Japan.

### Luminescence Imaging and CNS Disease Model

Tumor development in live mice was monitored by repeated noninvasive optical imaging of tumor-specific luciferase activity using the IVIS Lumina XR Imaging System (PerkinElmer), as previously described.^18^ Bioluminescence intensity was analyzed with Living Image 4.0 (PerkinElmer) by quantifying the peak photon flux within a selected region of interest (ROI). The signal was corrected for ROI area and CCD camera exposure time and was expressed as photons per second (photons/s).

### Leptomeningeal Carcinomatosis Model

Mouse scalps were sterilized with 70% ethanol, and PB302/Eluc cells (1 × 10^5^/50 μL) were injected into the leptomeningeal space between the external occipital protuberance and the first cervical vertebra using a 27-gauge needle.^19^ Three SHO mice were used to establish and validate the leptomeningeal carcinomatosis model.

### Brain Tumor Model

PB302/Eluc cells were inoculated into the brain as previously described.^15^ SHO mice were anesthetized with tribromoethanol. After sterilizing the scalp with 70% ethanol, a small hole was drilled into the skull 0.5 mm anterior and 3.0 mm lateral to the bregma using a dental drill. Tumor cells (1.5 × 10^5^/1.5 μL) were injected into the right striatum 3 mm below the brain surface using a 10-μL Hamilton syringe with a 26-gauge needle. The scalp was then closed using an AutoClip Applier (BD). Two SHO mice were used to establish and confirm the brain tumor model.

### Subcutaneous Tumor Model

PB302/Eluc intravenous (i.v.) LM cells (Supplementary Data 1A, blue dish), derived from leptomeningeal lesions, were injected subcutaneously into SHO mouse flanks at 5 × 10^6^ cells. After removal and mincing of the resulting subcutaneous tumor (Supplementary Data 1A, red arrowhead), cells were cultured to generate PB302/Eluc i.v. LM>SC cells (Supplementary Data 1A, orange dish). These cells were again injected subcutaneously at the same concentration. Tumor size and body weight were measured twice weekly, and tumor volume was calculated using the following formula: tumor volume (mm^3^) = (width^2^ × length)/2, measured using digital calipers. Two SHO mice were used to establish and validate the subcutaneous tumor model. Treatment experiments for the subcutaneous tumor model were also conducted using the same two SHO mice, both of which were assigned to the treatment group.

### Brain Metastasis Model

The PB302/Eluc cell suspensions (5 × 10^6^/100 μL) were prepared, and 100 μL was injected into the tail vein of mice. Two mice were used to establish the brain metastasis model.

### Statistical Analysis

Viability assay data were expressed as mean ± SD. Statistical significance was assessed using one-way analysis of variance and Spearman’s rank correlation with GraphPad Prism version 6.0 (GraphPad Software). A two-sided *p* value less than 0.05 was considered statistically significant.

### Informed Consent Statement

Consent for publication in print and online was obtained from the patient and their family.

## Results

### Patient Characteristics and Clinical Course

A 49-year-old female never-smoker with *RET* fusion-positive lung cancer sought treatment at the Kanazawa University Hospital for selective *RET* TKI selpercatinib. The patient’s condition included a primary tumor in the upper lobe of the left lung, multiple mediastinal lymph nodes, left pleural effusion, multiple brain metastases, and leptomeningeal carcinomatosis. Because of insufficient tissue, a commercially available gene profiling test could not be performed; therefore, the patient initially received carboplatin (700 mg/body), pemetrexed (830 mg/body), and pembrolizumab (200 mg/body). During this treatment, a computed tomography (CT)–guided biopsy was performed for genomic profiling using the Oncomine Dx Target Test, which confirmed the presence of *RET* fusion in the tumor sample, although the *RET* fusion partner was not identified in the report. The first-line therapy had limited effectiveness, prompting her attending physician to request the selective *RET* TKI selpercatinib through an expanded-access program, leading to a referral to the Department of Medical Oncology at Kanazawa University. On arrival at our institution, the patient presented with impaired consciousness and received 160 mg of selpercatinib via a gastric tube twice daily. A pleural effusion sample was collected for cell line establishment before selpercatinib initiation. On the following day, her consciousness improved, and she was able to take 160 mg of selpercatinib orally twice daily. One month after initiating selpercatinib, a drastic response was observed in intracranial and extracranial lesions (Fig. 1*C* and *D*).

### Fusion Partner Gene in PB302

**Figure 2:**
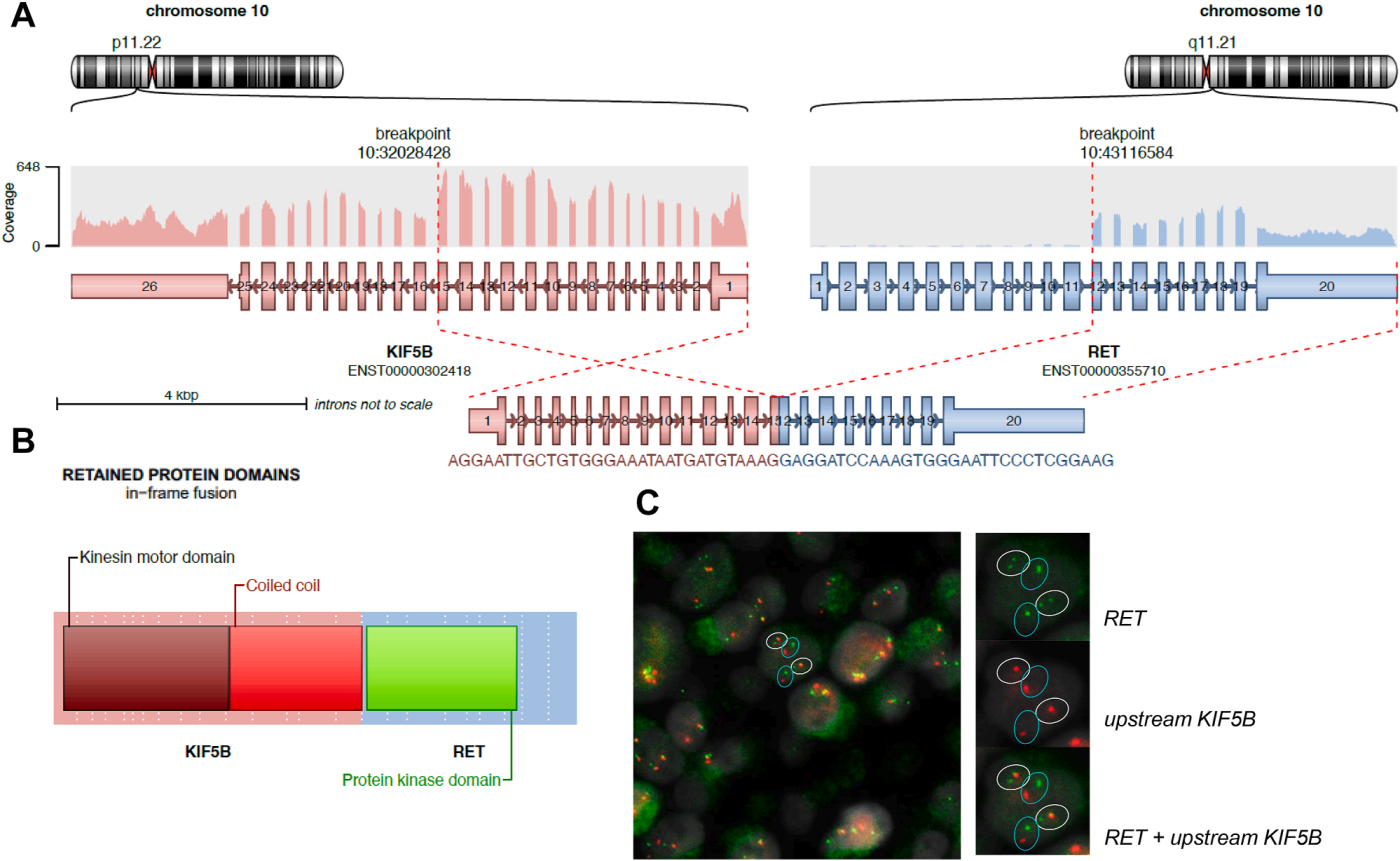
Detection of fusion genes in PB302 and their details. (*A*) The fusion partner gene, *KIF5B*, located on chromosome 10, fuses with the *RET* gene via inversion at each breakpoint. PB302 contains a *KIF5B-RET* fusion with the K15;R12 variant. (*B*) The truncated *RET* gene retains a tyrosine kinase domain, whereas the truncated *KIF5B* gene includes a kinesin motor domain and coiled-coil domain, which is crucial for dimerization. (*C*) As an example of a single cell, the white-outlined region represents the combination of *KIF5B-RET* (yellow) resulting from inversion and the remaining 5' *RET* (*RET-KIF5B*), whereas the green-outlined region represents the normal combination of *KIF5B* and *RET* on chromosome 10.

RNA sequencing results provided information on the chromosomal locations of each gene, including the fusion genes and the domains of these fusion genes (Fig. 2*A* and *B*). PB302 cells exhibited *KIF5B-RET* fusion, with the *KIF5B* breakpoint located in exon 15 and the *RET* breakpoint in intron 11. These variants were designated as K15 and R12 (Fig. 2*A* and *B*). In addition, the presence of *RET* fusion genes in PB302 was confirmed by FISH (Fig. 2*C*). The probe on the *KIF5B* side (red) was RP11-460H18, located approximately 60 kb upstream of *KIF5B*. The probe on the RET side (green) was RP11-351D16 and completely covered RET in the central region, which was approximately three times its length.

### Sensitivity of PB302 and Other RET Fusion–Positive Cell Lines to Selective RET Inhibitors

**Figure 3:**
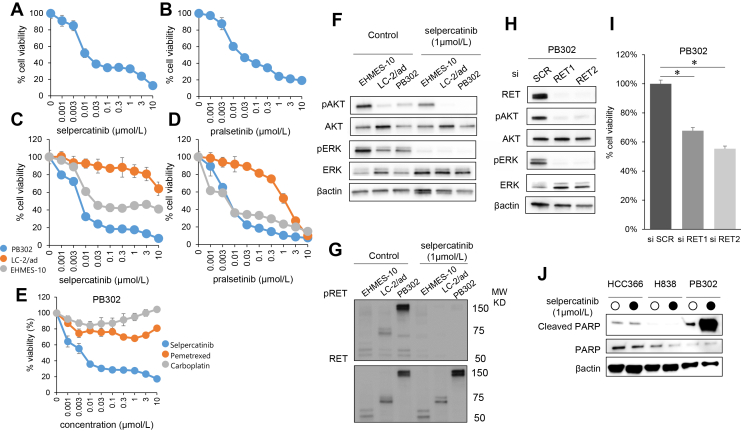
The survival of PB302 depends on signals from the *RET* fusion protein. (*A*, *B*) PB302 demonstrates sensitivity to selective *RET* TKIs. The effect of selpercatinib and pralsetinib on PB302 viability was determined using an MTT assay. Data are presented as the mean ± SD of three independent biological experiments, each performed in triplicate technical wells. (*C*, *D*) Compared with two positive control cell lines, EHMES-10 with *NCO4A-RET* and LC-2/ad with *CCDC6-RET*, PB302 exhibits the highest sensitivity to selective *RET* TKIs. The sensitivity of LC-2/ad, EHMES-10, and PB302 cells to selpercatinib or pralsetinib was determined using an MTT assay. Data are presented as the mean ± SD of three independent biological experiments, each performed in triplicate technical wells. (*E*) Sensitivity to each drug, including chemotherapeutic agents, in PB302 was determined using an MTT assay. Data are presented as the mean ± SD of three independent biological experiments, each performed in triplicate technical wells. (*F*, *G*) Tumor cells were treated with selpercatinib (1 μmol/L) for 4 hours and harvested; lysates were analyzed by Western blotting. Exposure to selpercatinib effectively blocked survival signals, reducing the phosphorylation of target molecules. The molecular weight of *NCO4A-RET* or *CCDC6-RET* ranges from 50 kDa to 75 kDa. (*H*, *I*) PB302 cells were transfected with *RET*-specific siRNA (siRET) or a control siRNA (siSCR). After 48 hours, cell lysates were harvested and assessed for protein expression by Western blotting. siRET resulted in loss of *RET* expression and decreased cell viability. Cell viability was then determined using an MTT assay. Data (mean ± SD) illustrated are representative of three independent experiments with similar results. ∗*p* < 0.05, as determined by the Student’s *t* test, compared with siSCR. (*J*) Expression of cleaved PARP after 48-hour exposure to selpercatinib (1 μmol/L) in HCC366, H838, and PB302. ● indicates selpercatinib exposure; 〇 indicates no drug exposure.

We evaluated the efficacy of selpercatinib and pralsetinib in three *RET*-fusion positive cell lines, including PB302 (Fig. 3*A*–*D*). LC-2/ad and EHMES-10 were selected as positive control cell lines because they harbor *RET* fusion and were expected to be sensitive to selective *RET* inhibitors. Surprisingly, LC-2/ad was not sensitive to these inhibitors, and repeated viability assays yielded consistent results. Regarding this phenomenon, Wei et al.^20^ reported that LC-2/ad demonstrated resistance to *RET* inhibitors when viability was assayed in 2D culture but had sensitivity when evaluated in 3D culture. In contrast, EHMES-10 demonstrated promising results and was previously reported to be sensitive to another *RET* inhibitor, alectinib, in vitro and in vivo.^21^ For PB302, the inhibition curves for selpercatinib and pralsetinib were sigmoid, with IC_50_ values of 5.876 and 3.966 nmol/L, respectively. As anticipated, PB302 cells were sensitive to selpercatinib and pralsetinib. In addition, the sensitivity of PB302 cells to carboplatin and pemetrexed was evaluated using the MTT assay. PB302 is a cell line established from pleural effusion obtained when the tumor had no response to these cytotoxic anticancer agents, and, as expected, no sensitivity to either drug was observed (Fig. 3*E*).

### Signal Changes and Apoptosis Observed With Selective RET Inhibitor Exposure in PB302 and the Effects of RET Knockdown

We assessed the impact of selpercatinib treatment on ERK and AKT signaling in EHMES-10, LC-2/ad, and PB302 cell lines. Selpercatinib (1 μmol/L) effectively reduced the phosphorylation of *RET*, AKT, and ERK in all three cell lines (Fig. 3*F*). Western blot analysis revealed that the molecular weight of phosphorylated *RET* in PB302 was approximately 150 kDa (Fig. 3*G*), which aligned with the molecular weight of the recombinant human *GST-KIF5B-RET* fusion protein (ab268707) produced by Abcam. We also investigated the effects of *RET* knockdown in PB302 cells using *RET*-specific siRNAs. Both siRNAs effectively reduced *RET* expression and PB302 cell viability (Fig. 3*H* and *I*). Furthermore, we evaluated the apoptosis status of HCC366, H383, and PB302 cells exposed to selpercatinib at a concentration of 1 μmol/L for 48 hours. Compared with wild-type HCC366 and H383, PB302 clearly demonstrated stronger expression of cleaved PARP (Fig. 3*J*).

### Establishment of CNS Disease Models and Subcutaneous Tumor Model

**Figure 4:**
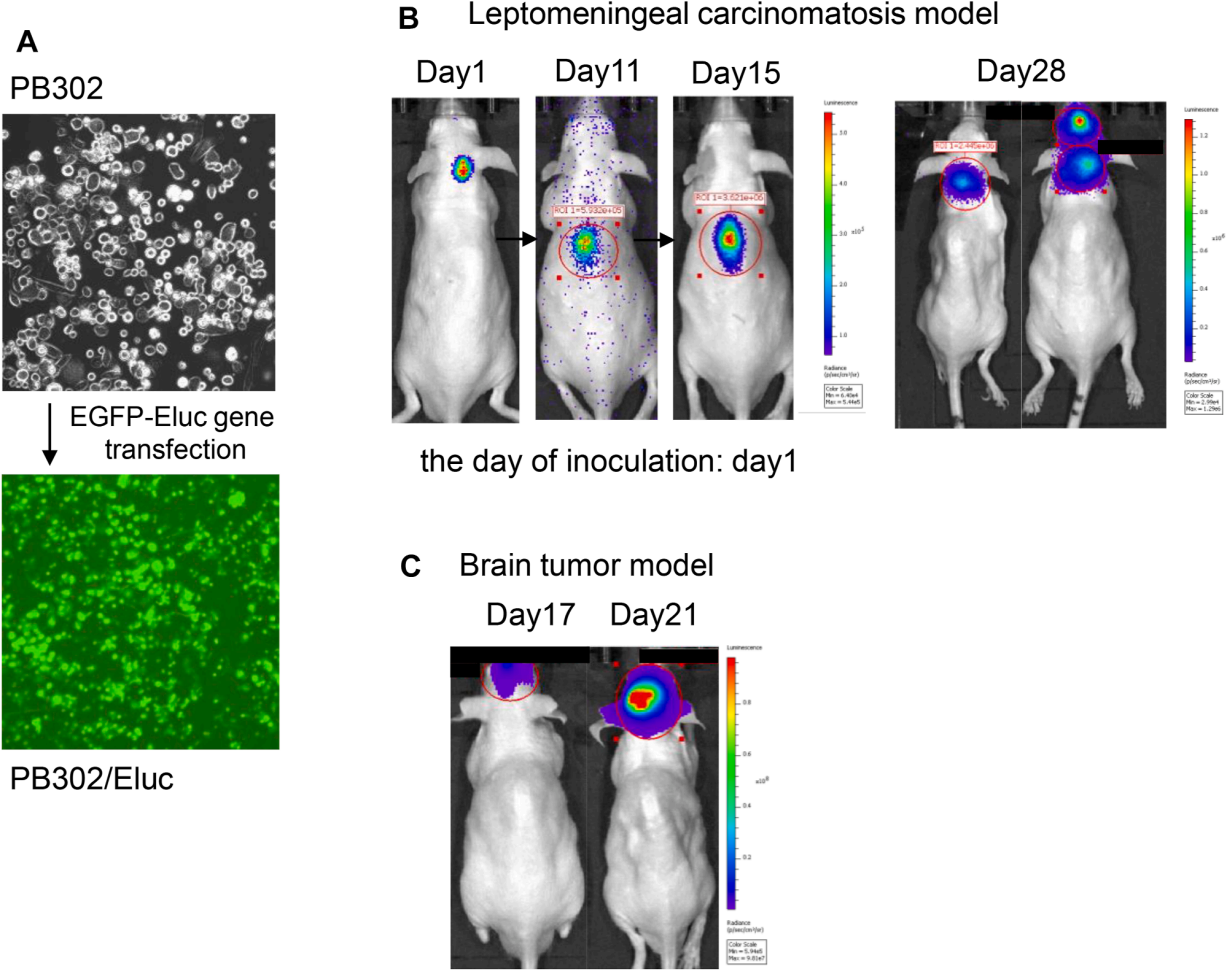
PB302/Eluc inoculated into the leptomeningeal space or the right brain grows and is detectable as luminescence. (*A*) Fluorescent imaging confirms successful transfection of the EGFP-Eluc gene into PB302. (*B*) Day 1 is defined as the day of inoculation. Over time, luminescence intensifies in the leptomeningeal carcinomatosis model (*n* = 3). (*C*) The brain tumor model demonstrates a similar increase in luminescence (*n* = 2).

PB302 cells, when inoculated into the flanks of SHO mice, were unable to grow or enlarge. Given the patient’s history of aggressive leptomeningeal carcinomatosis and multiple brain metastases, we hypothesized that PB302 cells may grow only in the CNS. To test this, we transfected PB302 with EGFP-Eluc to create PB302/Eluc (Fig. 4*A*), enabling the detection of luminescence that would indicate the presence of viable lesions in the CNS models. As anticipated, we successfully established models of leptomeningeal carcinomatosis and brain tumors (Fig. 4*B* and *C*). Luminescent lesions were collected and cultured in a 10-cm dish with 10 mL of medium, resulting in a new cell line named PB302/Eluc i.v. LM (Supplementary Data 1A, blue dish). Referring to the phenomenon in which glioblastoma cell lines establish and enlarge in the subcutaneous tissue,^22^^,^^23^ we established a subcutaneous tumor model by injecting PB302/Eluc i.v. LM cells into the flanks of SHO mice (Supplementary Data 1A, red arrowhead). After removing and mincing the subcutaneous tumor, we cultured it in a 10-cm dish with 10 mL of medium, leading to the development of a new cell line, PB302/Eluc i.v. LM>SC (Supplementary Data 1A, orange dish). Following the concept of in vivo selection, we injected PB302/Eluc i.v. LM>SC into the flank of SHO mice, which required 4 days for a subcutaneous tumor to form (Supplementary Data 1A, dashed red circle). This confirmed the reproducibility of establishing a subcutaneous tumor model using PB302/Eluc i.v. LM**>**SC.

### Selpercatinib Was Effective in Mouse Subcutaneous Tumors Created Using PB302/Eluc i.v. LM>SC

Treatment with selpercatinib (10 mg/kg) twice daily (BID) was initiated when the subcutaneous tumor reached approximately 200 mm^3^. The tumor rapidly shrank, and selpercatinib was found to be effective in vivo (Supplementary Data 1B).

### PB302/Eluc Has Metastatic Potential

Metastasis is present at diagnosis in a significant fraction of *RET* fusion-positive lung cancers, indicating the metastatic nature of *RET*-driven lung tumors. Using the common tail-vein injection method, PB302/Eluc was confirmed to metastasize to the brain (Supplementary Data 1C). It took more than 2 months for brain metastases to form after the i.v. injection of PB302/Eluc.

### Pathology of Leptomeningeal Carcinomatosis Model

**Figure 5:**
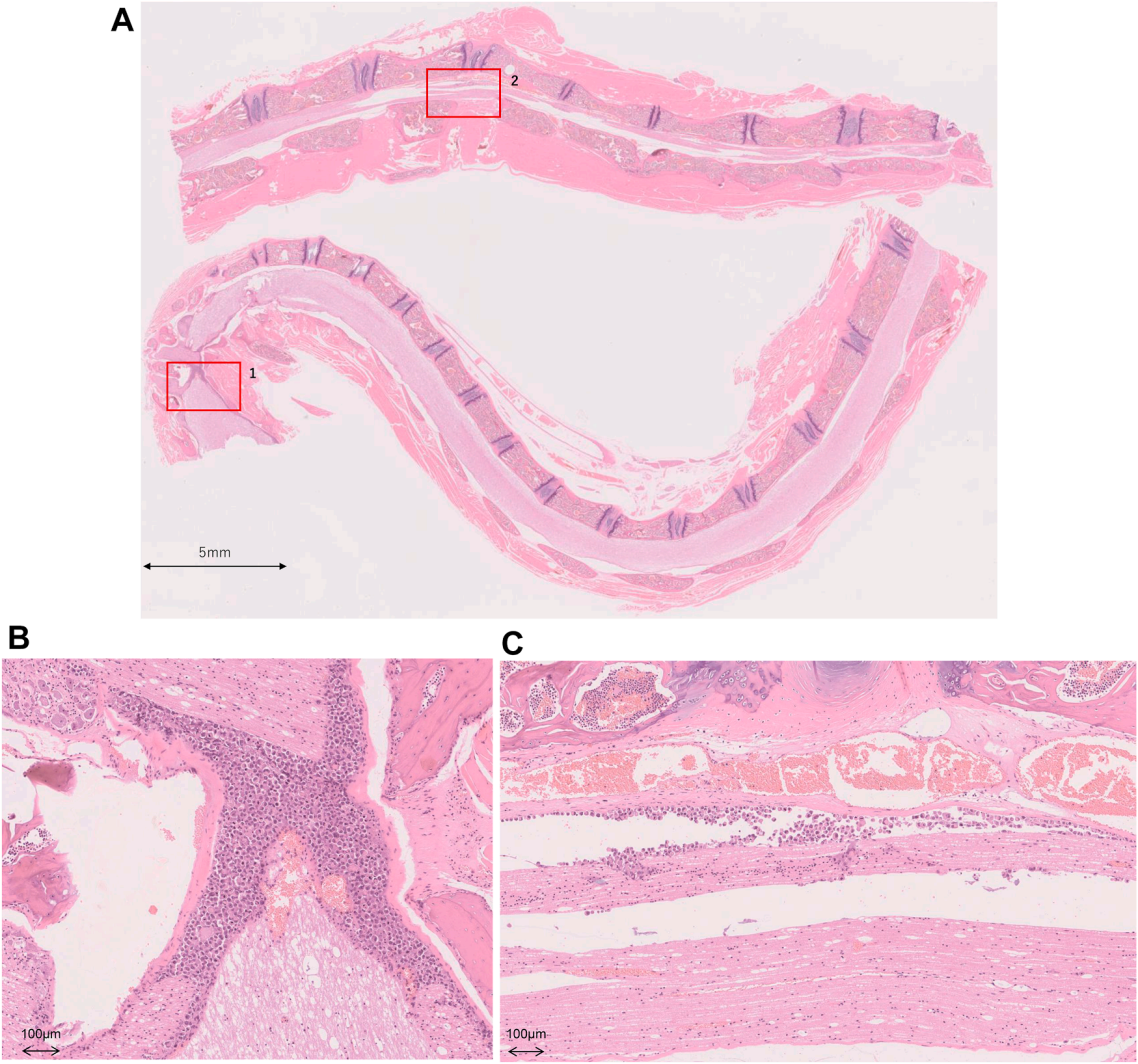
Histopathology of sagittal sections of the whole spine collected from the leptomeningeal carcinomatosis model. (*A*) Overview of the spine, with areas of interest marked by red squares 1 (cranial) and 2 (caudal). (*B*) High-power view of the cranial side. (*C*) High-power view of the caudal side. Cancer cells are attached to the spinal cord.

All vertebrae were sectioned sagitally to obtain a comprehensive view. In the lower sections, the left and right sides represented the cranial and caudal ends, respectively (Fig. 5*A*). The cranial side, closer to the injection site, exhibited a dense accumulation of cancer cells (Fig. 5*B*). Conversely, the caudal side displayed a sparse distribution of cancer cells (Fig. 5*C*).

### Histopathologic Characteristics of a Subcutaneous Tumor of PB302/Eluc i.v. LM>SC

The histology of the patient’s original tumor exhibited a solid pattern of fibrosis in the stromal tissue, and immunohistochemical staining result was negative for napsin A and TTF-1 (Supplementary Data 2A and C). In the subcutaneous tumor of the PB302/Eluc i.v. LM>SC group, papillary and tubular structures were not observed (Supplementary Data 2D), which made it appear similar to the original tumor. Immunohistochemical analysis revealed that the subcutaneous tumor was negative for napsin A (352A-74-RUO) and TTF-1 (MOB285-01) (Supplementary Data 2E and F). The subcutaneous tumor tested positive for RET (HPA008356) (Supplementary Data 2G and H).

### Mutational Signature Analysis

Mutational signature analysis was performed on PB302 using SigProfiler, which identified a single novel signature, SBS96A. Comparative analysis with COSMIC signatures revealed that SBS96A demonstrated strong similarity to the COSMIC signature groups SBS5 (30%–35%), SBS39 (25%–30%), SBS8 (20%–25%), SBS9 (10%–15%), and SBS98 (5%–10%) (Supplementary Data 3).

## Discussion

Several *RET* fusion-positive cell lines derived from clinical specimens have been reported. To compare these with PB302, we evaluated its characteristics (Table 1). LC-2/ad was the first *RET* fusion-positive lung cancer cell line reported and carries the second most common fusion partner, CCDC6. It is also the only commercially available *RET* fusion-positive cell line.^12^ In 2D cultures using flat-bottomed 96-well plates, LC-2/ad has been reported to demonstrate resistance to selective *RET* inhibitors,^17^ and in our experiments, it was also insensitive to these inhibitors. In contrast, the PB302 cell line we established demonstrated sensitivity to selective *RET* inhibitors even in 2D cultures (Fig. 3). Although we could not establish a subcutaneous tumor model with our LC-2/ad line, Yamada et al. achieved this and are conducting therapeutic experiments; however, no metastatic models have been reported.

**Table 1:** Comparison of Reported *RET* Fusion-Positive Lung Cancer Cell Lines and Our Patient-Derived *RET* Fusion Cell Line

Cell Line	Fusion Partner	Frequency of Fusion Partner	Cell Line Characteristics	IC_50_ of Selective *RET* Inhibitor (nmol/L)	Subcutaneous Tumor Model	Examination of Metastatic Model	Note
LC-2/ad	CCDC6	∼10%–25%	Adherent	>10,000 (selpercatinib) 1119 (pralsetinib)	Controversial^13^^,^^26^	-	Commercially available
CUTO22	KIF5B	∼70%–90%	Suspension	20 ± 10 (pralsetinib)	Impossible	-	Established at University of Colorado School of Medicine^22^
CUTO32	KIF5B	∼70%–90%	Suspension	342 ± 221 (pralsetinib)	Possible	-	
CUTO42	EML4	Rare	Mixed suspension and adherent	11 ± 4 (pralsetinib)	Possible	-	
ECLCB5	TRIM33	Rare	Adherent	Not revealed	Not revealed	Brain metastasis model	Established at Memorial Sloan Kettering Cancer Center^23^
PB302	KIF5B	∼70%–90%	Adherent	5.876 (selpercatinib) 3.966 (pralsetinib)	Possible	Central nervous disease model	Established at Kanazawa University

The University of Colorado School of Medicine established three *RET* fusion cell lines.^13^ Among them, CUTO42 is sensitive to pralsetinib and forms subcutaneous tumors in immunodeficient mice. However, CUTO42 grows in both adherent and suspension cultures, complicating handling. Furthermore, its *RET* fusion partner, EML4, is rare and not representative of most *RET* fusion-positive lung cancers.^24^

The Memorial Sloan Kettering Cancer Center established the ECLCB5 cell line harboring the *TRIM33-RET* fusion.^14^ This line was used in a brain metastasis model presented at ASCO 2022. However, no subcutaneous tumor model has been reported, and the *TRIM33-RET* fusion is rare, limiting its generalizability. The institute also used the LUAD-0002AS1 cell line derived from multiple passages of patient-derived xenograft tissues with *RET* fusion, although maintaining such models is costly.

In PB302, the fusion partner *KIF5B* was the most common, with a breakpoint at exon 15 and *RET* at intron 11. This variant, referred to as K15;R12, is the most frequent among seven *KIF5B-RET* fusion variants, accounting for 75% of patients with *KIF5B-RET*.^25^ In cell viability assays, PB302 demonstrated the expected sensitivity to the selective *RET* inhibitors selpercatinib and pralsetinib. We also established subcutaneous tumor and CNS disease models. Selpercatinib was effective in the PB302/Eluc i.v. LM>SC subcutaneous tumor model (Supplementary Data 1B). Morphologically and immunohistochemically, mouse subcutaneous tumors resembled the original bronchoscopic specimens (Supplementary Data 2). Luminescence imaging revealed that tail-vein injection of PB302/Eluc enabled brain engraftment and growth (Supplementary Data 1C), indicating metastatic potential.

PB302 enables modeling of CNS and subcutaneous tumors, providing a platform to study drug resistance. Future studies should assess long-term resistance development and genetic alterations. PB302 may also be valuable for developing next-generation selective *RET* inhibitors and assessing their efficacy across tumor microenvironments. To further validate the characteristics of PB302, whole-genome sequencing was performed. The results are available upon request (Supplementary Data 4, Table 1A–D). Mutational signature analysis identified a single signature, SBS96A, resembling COSMIC signature SBS5, which is commonly observed in patients with lung cancer with driver gene mutations, including *RET* fusion genes, in nonsmokers.^27^

PB302 has some limitations. First, it was not established from treatment-naive pleural fluid at diagnosis; prior chemoimmunotherapy may have influenced tumor cell drug sensitivity and other characteristics. Furthermore, a subcutaneous tumor model required labor-intensive in vivo selection. The subcutaneous tumor experiment involved only two mice and was exploratory, limiting the interpretation of the findings. The *RET* fusion gene is a tumor-agnostic target, and selpercatinib is available under health insurance coverage in Japan. We hypothesized that, as solid tumors harboring *RET* fusion genes, lung cancer and malignant pleural mesothelioma could be treated as equivalent. However, because the frequency of *RET* fusion positivity and the clinical characteristics observed in lung cancer do not apply to mesothelioma, the use of EHMES-10 in this study represents a limitation. Despite these limitations, PB302 is expected to contribute to the development of next-generation selective *RET* inhibitors. A patent application for this cell line is currently in progress (patent number: 2023-133367).

In conclusion, PB302, which harbors a *KIF5B-RET* fusion, demonstrates (1) significant sensitivity to selective *RET* inhibitors in vitro and in vivo; (2) subcutaneous growth in immunodeficient mice; and (3) the ability to establish CNS disease models. These features make PB302 valuable for in vivo experiments to induce drug resistance in extracranial and intracranial lesions and for studying resistance mechanisms.

## CRediT Authorship Contribution Statement

**Nishiyama Akihiro:** Conceptualization, Writing - original draft preparation, Writing - review & editing.

**Shigeki Sato:** Investigation, Review.

**Hiroyuki Sakaguchi:** Review.

**Hiroshi Kotani:** Review.

**Kaname Yamashita:** Review.

**Koushiro Ohtsubo:** Review.

**Shigeki Nanjo:** Methodology and Review.

**Seiji Yano:** Review.

**Keishi Mizuguchi:** Review.

**Hiroko Ikeda:** Review.

**Makiko Yamaguchi:** Investigation and Review.

**Tamotsu Ishizuka:** Review.

**Satoko Baba:** Investigation.

**Kengo Takeuchi:** Investigation.

**Shinji Takeuchi:** Writing - reviewing & editing.

## Disclosure

Dr. Nanjo received a speaker fee from AstraZeneca. Dr. Yano obtained research grants and speaking honoraria from Eli Lilly, Japan. Dr. Ishizuka received research grants from Eli Lilly and speaker fees and honoraria from AstraZeneca. Dr. Kengo Takeuchi received contracts from Meiji Seika Pharma and Nippon Shinyaku; royalties from Sysmex, Otsuka Pharmaceutical, REALM IDx, and Nichirei Bioscience; consulting fees from Nichirei Bioscience, Meiji Seika Pharma, and Nippon Shinyaku; and speaker bureaus from Kyowa Kirin, Chugai, SymBio Pharmaceuticals, AstraZeneca, Eisai, and Janssen Pharmaceuticals. Dr. Shinji Takeuchi received research grants from Eli Lilly, 10.13039/100002429Amgen, and Chugai Pharmaceutical, Co., and speaker fees from Guardant Health Japan, Eli Lilly, and Chugai Pharmaceutical Co. The remaining authors declare no conflict of interest.

## Supplementary Data

Supplementary Table 4

JTO_CRR_PB302_Supplementary_Figures_with_captions

## References

[1] Takahashi M., Ritz J., Cooper G.M. (1985). Activation of a novel human transforming gene, ret, by DNA rearrangement. Cell.

[2] Takahashi M. (2022). RET receptor signaling: function in development, metabolic disease, and cancer. Proc Jpn Acad Ser B Phys Biol Sci.

[3] Mulligan L.M. (2014). RET revisited: expanding the oncogenic portfolio. Nat Rev Cancer.

[4] Saito M., Shiraishi K., Kunitoh H., Takenoshita S., Yokota J., Kohno T. (2016). Gene aberrations for precision medicine against lung adenocarcinoma. Cancer Sci.

[5] Kohno T., Ichikawa H., Totoki Y. (2012). *KIF5B-RET* fusions in lung adenocarcinoma. Nat Med.

[6] Gautschi O., Park K., Solomon B.J. (2025). Selpercatinib in RET fusion-positive non-small cell lung cancer: final safety and efficacy, including overall survival, from the LIBRETTO-001 phase I/II trial. J Clin Oncol.

[7] Wirth L.J., Sherman E., Robinson B. (2020). Efficacy of selpercatinib in RET-altered thyroid cancers. N Engl J Med.

[8] Drilon A., Subbiah V., Gautschi O. (2023). Selpercatinib in patients with RET fusion-positive non-small cell lung cancer: updated safety and efficacy from the registrational LIBRETTO-011 phase I/II trial. J Clin Oncol.

[9] Subbiah V., Wolf J., Konda B. (2022). Tumor-agnostic efficacy and safety of selpercatinib in patients with RET fusion-positive solid tumours other than lung or thyroid tumours (LIBRETTO-011): a phase 1/2, open-label, basket trial. Lancet Oncol.

[10] Subbiah V., Shen T., Terzyan S.S. (2021). Structural basis of acquired resistance to selpercatinib and pralsetinib mediated by non-gatekeeper RET mutations. Ann Oncol.

[11] Waliany S., Cooper A.J., Liu S.V. (2026). Landscape of genomic mechanisms of resistance to selective RET inhibitors in RET-altered solid tumors: analysis of the RETgistry global consortium. Clin Cancer Res.

[12] Matsubara D., Kanai Y., Ishikawa S. (2012). Identification of CCDC6-RET fusion in the human lung adenocarcinoma cell line, LC-2/ad. J Thorac Oncol.

[13] Schubert L., Le A.T., Estrada-Bernal A. (2021). Novel human-derived RET fusion NSCLC cell lines have heterogeneous responses to RET inhibitors and differential regulation of downstream signaling. Mol Pharmacol.

[14] Hayashi T., Odintsov I., Smith R.S. (2020). RET inhibition in novel patient-derived models of RET-fusion positive lung adenocarcinoma reveals a role for MYC upregulation. Dis Model Mech.

[15] Nanjo S., Nakagawa T., Takeuchi S. (2015). In vivo imaging models of bone and brain metastases and pleural carcinomatosis with a novel human EML4-ALK lung cancer cell line. Cancer Sci.

[16] Corti G., Buzo K., Berrino E. (2024). Prediction of homologous recombination deficiency identifies colorectal tumors sensitive to PARP inhibition. NPJ Precis Oncol.

[17] Takeuchi K., Soda M., Togashi Y. (2012). RET, ROS1 and ALK fusions in lung cancer. Nat Med.

[18] Takeuchi S., Fukuda K., Arai S. (2016). Organ-specific efficacy of HSP90 inhibitor in multiple-organ metastasis model of chemorefractory small cell lung cancer. Int J Cancer.

[19] Nanjo S., Arai S., Wang W. (2017). MET copy number gain is associated with gefitinib resistance in leptomeningeal carcinomatosis of EGFR-mutant lung cancer. Mol Cancer Ther.

[20] Wei X., Uchibori K., Kondo N. (2024). MIG6 loss increased RET inhibitor tolerant persister cells in RET-rearranged non-small cell lung cancer. Cancer Lett.

[21] Arai S., Kita K., Tanimoto A. (2017). In vivo and in vitro anti-tumor activity of alectinib in tumor cells with NCOA4-RET. Oncotarget.

[22] Fujikawa A., Sugawara H., Tanaka T. (2017). Targeting PTPRZ inhibits stem cell-like properties and tumorigenicity in glioblastoma cells. Sci Rep.

[23] Sasame J., Ikegaya N., Kawazu M. (2022). HSP90 inhibition overcomes resistance to molecular targeted therapy in BRAFV600E-mutant high-grade glioma. Clin Cancer Res.

[24] Guo R., Schreyer M., Chang J.C. (2019). Response to selective RET inhibition with LOXO-292 in a patient with RET fusion-positive lung cancer with leptomeningeal metastases. JCO Precis Oncol.

[25] Ferrara R., Auger N., Auclin E., Besse B. (2018). Clinical and translational implications of RET rearrangements in non-small cell lung cancer. J Thorac Oncol.

[26] Kang C.W., Jang K.W., Sohn J. (2015). Antitumor activity and acquired resistance mechanism of dovitinib (TKI258) in RET-rearranged lung adenocarcinoma. Mol Cancer Ther.

[27] Moorthi S., Paguirigan A., Itagi P. (2024). The genomic landscape of lung cancer in never-smokers from the Women’s Health Initiative. JCI Insight.

